# *In Vivo* Detection of Age-Related Tortuous Cerebral Small Vessels using Ferumoxytol-enhanced 7T MRI

**DOI:** 10.14336/AD.2023.1110-1

**Published:** 2024-08-01

**Authors:** Zhe Sun, Chenyang Li, Thomas W Wisniewski, E. Mark Haacke, Yulin Ge

**Affiliations:** ^1^Department of Radiology, NYU Grossman School of Medicine, New York, NY, USA.; ^2^Vilcek Institute of Graduate Medical Sciences, NYU Grossman School of Medicine, New York, NY, USA.; ^3^Department of Neurology, NYU Grossman School of Medicine, New York, NY, USA.; ^4^Department of Radiology, Wayne State University, Detroit, MI, USA.

**Keywords:** cerebral small vessel disease, ultra-small superparamagnetic iron oxide, tortuous vessel, medullary artery, MRI

## Abstract

Histopathological studies suggest that cerebral small vessel tortuosity is crucial in age-related blood flow reduction and cellular degeneration. However, in vivo evidence is lacking. Here, we used Ferumoxytol-enhanced 7T MRI to directly visualize cerebral small vessels (<300 µm), enabling the identification of vascular tortuosity and exploration of its links to age, tissue atrophy, and vascular risk factors. High-resolution 2D/3D gradient echo MRI at 7T enhanced with Ferumoxytol, an ultrasmall superparamagnetic iron oxide (USPIO), was obtained and analyzed for cerebral small medullary artery tortuosity from 37 healthy participants (21-70 years; mean/SD: 38±14 years; 19 females). Tortuous artery count and tortuosity indices were compared between young and old groups. Age effects on vascular tortuosity were examined through partial correlations and multiple linear regression, adjusting for sex, body mass index (BMI), blood pressure (BP), and other vascular risk factors. Associations between tortuous medullary arteries and tissue atrophy, perivascular spaces (PVS), and white matter (WM) hyperintensities were explored. Age and BMI, rather than BP, showed positive correlations with both tortuous artery count and tortuosity indices. A significant correlation existed between the number of tortuous arteries and WM atrophy. WM lesions were found in proximity to or at the distal ends of tortuous medullary arteries, especially within the deep WM. Moreover, the elderly population displayed a higher prevalence of PVS, including those containing enclosed tortuous arteries. Leveraging the blooming effect of Ferumoxytol, 7T MRI excels in directly detecting cerebral small arterial tortuosity in vivo, unveiling its associations with age, BMI, tissue atrophy, WMH and PVS.

## Introduction

Cerebral small vessels ranging from 50 to 300 μm in diameter, particularly medullary arteries (MAs) responsible for deep white matter (WM) blood supply, undergo age-related vascular degenerative processes, leading to corkscrew-like configurations [1, 2]. These alterations increase susceptibility to ischemic injury in the deep WM due to MAs' small size and lack of anastomoses. Postmortem histological studies have documented age-related morphological changes of these MAs [3, 4]. These changes are macroscopically evident on MRI through the presence of white matter hyperintensities (WMHs) or leukoaraiosis on T2 or fluid attenuation inversion recovery (FLAIR) [5]. However, direct in vivo visualization is currently lacking. Vascular degeneration typically initiates with wall remodeling due to factors including mechanical stress from pulsatile blood flow, inflammation, collagen deposition, and elastin degradation [6, 7]. These changes of small penetrating arteries may manifest considerably earlier than pronounced clinical symptoms. Consequently, the tortuosity of small vessels could potentially serve as an early marker for brain aging, formation of WMHs, and cognitive dysfunction associated with cerebral small vessel disease (CSVD) [8].

T2-weighted MR images occasionally exhibit the prominent perivascular spaces (PVSs) which are often assumed to coexist with the MAs [9]. The degenerative MAs with corkscrew appearance have only been identified within PVS in postmortem studies [10, 11]. While ultrasound and MR angiography studies have effectively revealed age-related vessel tortuosity in large elastic arteries [12, 13], the challenge remains to in vivo detect MAs and understand their relationship with PVS [14]. By exploiting the blooming effects and high susceptibility contrast offered by ultrasmall superparamagnetic iron oxide (USPIO) in conjunction with high-resolution T2*-weighted gradient echo (GRE) or susceptibility-weighted imaging (SWI), the visualization of small vessels at a microscopic scale (50-100μm) becomes achievable [15, 16]. Precisely evaluating changes in vessel tortuosity at this micro-level holds critical significance for early assessment of vascular risks and gaining deeper insights into the vascular factors contributing to CSVD in the elderly.

We hypothesize that age is a critical factor that facilitates the vascular degeneration linked to twisted appearance of MAs, a phenomenon that has not been previously demonstrated in vivo. This study aims to directly evaluate the tortuosity of small vessels concerning normal aging process and its resultant white matter degenerative changes. Utilizing USPIO-enhanced 7T MRI, we examined small vessel tortuosity in cognitively normal individuals. Qualitative and quantitative analyses of vascular tortuosity were undertaken to explore potential associations between tortuosity and variables like age, body mass index (BMI), tissue atrophy, and perivascular space (PVS). Moreover, we delved into the spatial connection between tortuous small arteries and ischemic white matter lesions.

## MATERIALS AND METHODS

### Participants

After obtaining approval from the review board, a total of 41 subjects were enrolled from the NYU Langone Health Center and written informed consents from each individual were obtained. Participants underwent screening and had no contraindications to MRI scan (pacemaker, implanted metallic objects, and claustrophobia et al). All subjects were generally healthy without documented medical conditions such as neurodegenerative disorders, cognitive impairment, brain injury, tremors, or medication usage that could potentially influence cognitive function. They were all right-handed native English speakers with Mini-Mental State Exam (MMSE) score of 26 or higher. Before the MRI scans, blood pressure (BP) and body mass index (BMI) were measured. Four participants were excluded due to motion artifacts or incomplete scans. And the final analysis comprised 37 participants (age range: 21-70 years; mean/SD: 38 ± 14 years old; 19 females).

### Imaging Protocol

All subjects underwent scanning on a 7T MRI scanner (MAGNETOM Siemens) with a 32-channel head coil. A low dose of Ferumoxytol (Feraheme, AMAG Pharmaceuticals, Inc. Waltham, MA), an FDA approved off-label iron-based contrast agent for treating iron deficiency anemia, was administered. Following the recommendations provided in the package insert, Ferumoxytol was administered via a gradual infusion (i.e., 15-20 minutes) into the antecubital vein to minimize the risk of anaphylactic reactions. To accommodate the specific contrast agent volume, the intravenous administration rate was between 150-200 ml/h, ensuring a delivery time of 18-20 minutes. The Ferumoxytol solution was mixed with 0.9% sodium chloride to achieve a final concentration of 2-3 mg/kg, which is approximately a quarter of therapeutic dosage [17]. Before the Ferumoxytol administration, 3D T1-weighted magnetization-prepared rapid acquisition of gradient echo (MPRAGE), axial T2*-weighted 2D gradient echo (GRE) and adapted 3D dual-echo GRE data with first echo flow compensated in slice/read direction were acquired. During the Ferumoxytol infusion, 2D T2-weighted turbo spin echo (TSE); 2D FLAIR; axial 2D GRE data were acquired; After the infusion, 3D dual-echo GRE data were acquired. Imaging parameters were as follows: 1) T1-weighted MPRAGE: repetition time/echo time (TR/TE) = 2300/3.24 ms, flip angle (FA) = 9°, voxel size = 1×1×1 mm^3^; 2) 2D T2-weighted TSE: TR/TE = 8000/90ms, FA=94°, voxel size = 0.8×0.8×2 mm^3^; 3) 2D FLAIR: TR/TE = 8000/90 ms, inversion time (TI)=2500ms, FA=130°, voxel size=1.3×1.0×2mm^3^; 4) Axial T2*-weighted 2D GRE: TR/TE=1250/25ms, FA=40°, voxel size=0.25×0.25×2 mm^3^, scan time = 6 min 58 sec, slice number = 30, bandwidth = 80 Hz/pixel; 5) 3D dual-echo GRE: TR/TE1/TE2 = 22/7.5/15 ms, FA=12°; voxel size = 0.25×0.25×1mm^3^, scan time = 14 min 16 sec, slice number = 96, bandwidth = 170 Hz/pixel. In order to reduce motion artifact, we positioned cushions between the subject's head and the head coil, which also improved comfort during the high-resolution scans. Furthermore, to mitigate image inhomogeneities that become pronounced at ultra-high field MRI, we first performed a low-resolution RF transmit/B1+ map to optimize transmitter voltage and correct for inhomogeneities. Finally, we performed manual shimming by adjusting gradients of X, Y, and Z directions with multiple iterations and then applied these calibrations to each sequence.

### MRI Data Processing

We processed 2D- and 3D GRE MRI data using in-house MATLAB (MathWorks, Natick, MA) scripts following the established processing flow [18, 19]. To distinguish arteries from veins, MR arteriogram (MRA) and MR venogram (MRV) maps were synthesized based on pre- and post-Ferumoxytol 3D dual echo GRE [20]. The pre-Ferumoxytol MRA map (MRA_nl_) was calculated to highlight the primary or large arterial tree structures, particularly the larger artereis with diameters exceeding 500μm. This was accomplished using a nonlinear subtraction of the long TE (TE2) from the short TE (TE1) of the pre-Ferumoxytol 3D-GRE magnitude data [19, 21]. The MRV map was calculated by dividing the post-Ferumoxytol TE1 magnitude data by the pre-Ferumoxytol TE1 magnitude data [17]. The synthesized MRA and MRV maps served as references to determine whether these larger vessels had arterial or venous origins. By carefully tracking the vascular trees from the larger to smaller vessels, we were able to distinguish small arteries (<300 μm) from small veins. Subsequently, these small arteries, which were exclusively visible in post-Ferumoxytol 3D-GRE images, were subjected to manual segmentation. The frangi-vesselness filter was then applied to the synthesized maps to highlight the origins of the vessel trunks [22]. Maximum intensity projection (MIP) and minimum intensity projection (mIP) of respective MRA and MRV were employed to visualize the vascular continuity.

After bias field correction, the MPRAGE images underwent segmented using FMRIB Software Library (FSL, Oxford, UK) [23] to quantify brain volume. The ratios of gray matter (GM) and WM relative to intracranial volume were calculated to indicate brain atrophy. FLAIR images were linearly registered to the post-Ferumoxytol SWI images using a six-parameter rigid transformation to reveal the spatial relationship between WMHs and tortuous small arteries.

**Figure 1.**
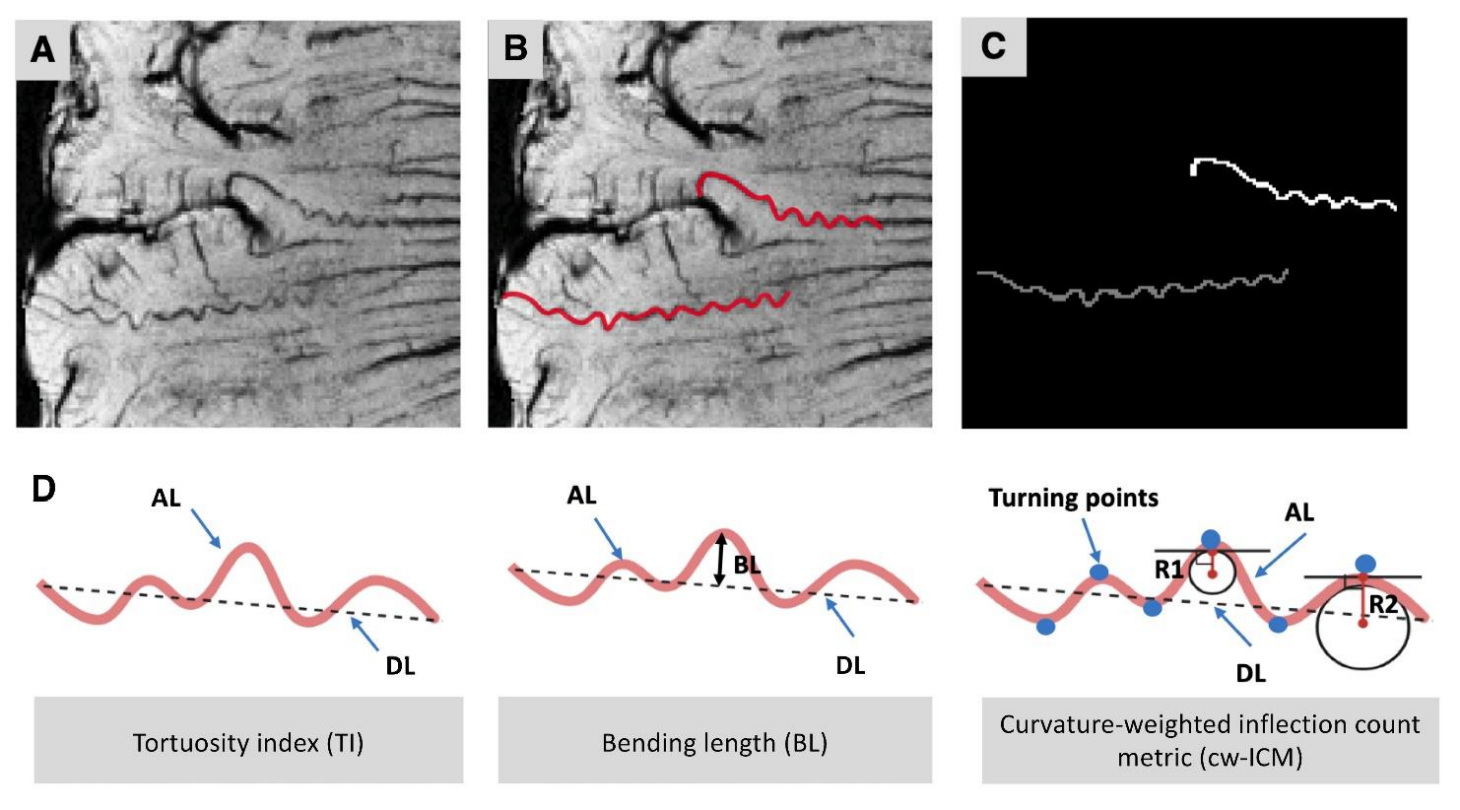
**Quantitative tortuosity measurements of the medullary artery**. (**A**) Post-contrast susceptibility-weighted imaging (SWI) data with minimum intensity projection of four (mIP = 4) clearly showed tortuous cerebral small arteries. (**B**) Two different tortuous small arteries were manually segmented assisted with ITK-SNAP toolbox (red). (**C**) Binary masks of the two vessels of interest (VOIs) were acquired for further centerline tracking and morphological analyses. (**D**) Quantitative morphological measurements include tortuosity index (TI), bending length (BL), and curvature-weighted inflection count metric (cw-ICM). TI = actual length (AL) / direct length (DL); BL is the maximum perpendicular distance between AL and DL; cw-ICM = maximum curvature (i.e., ) × turning points × TI.

### Vascular Tortuosity Measurements

The number of tortuous vessels was determined as the average count across all 2D GRE slices, benefiting from the higher T2 contrast differentiation among vessels, WM, and CSF. To accommodate scan duration and specific absorption rate (SAR) constraints at 7T, as well as the specific focus on MAs in this study, we utilized a 2-mm-thick 2D-GRE with 30 slices for imaging acquisition, providing a comprehensive coverage of the periventricular region where tortuous MAs are frequently observed. To ensure uniform brain coverage, the center slice was positioned at the lower edge of the corpus callosum body, aligned with the AC-PC line. To ensure sensitivity and consistency, we identified tortuous vessels by the corkscrew-like appearance, typically characterized by five or more turns. This determination was based on our observation that such vessels typically display a tortuosity index exceeding 1.05. Referring to the anatomical information from 2D-GRE data, we selected 8-10 slices from the 3D SWI image data, spanning 8-10mm and encompassing the periventricular region for the purpose of simplifying the manual segmentation procedure. Tortuous small arteries were manually delineated using the ITK-SNAP toolbox [24] on these slices, and the obtained vessel of interests (VOIs) were then subject to automatic centerline tracking and morphological analyses (Fig. 1A-C).

We computed various quantitative tortuosity measurements, including tortuosity index (TI), bending length (BL), and curvature-weighted inflection count metric (cw-ICM) (Fig. 1D). For each measurement, we computed the average values across various vessel segments within all selected slices for each subject. TI quantifies how much a blood vessel's trajectory curves compared to the straight line between its start and end points. It is computed by dividing the vessel's actual length (AL) by the linear distance between its endpoints, known as the direct length (DL). BL represents the greatest perpendicular distance between the vessel segment and the line connecting its starting and ending points. This parameter reflects how much the vessel's path deviates from a straight line [25]. Additionally, the calculation of cw-ICM integrates the maximum curvature along the vessel's course with an inflection count metric (ICM) [26]. Curvature at a specific point is calculated as the reciprocal of the radius of the circle that provides the best fit to the curve at that point, as illustrated using radii R1 and R2 in Fig. 1D. High curvature values with corresponding small radii indicate sharp bends and a higher degree of tortuosity. The cw-ICM calculation comprehensively considered factors such as the number of turning points, tortuosity index, and the curvature of these turning points, providing a comprehensive reflection of the overall vascular tortuosity.

To determine the correlation between enclosed tortuous arteries and the adjacent PVS, we quantified both the total number of the visible PVS and the number of PVS containing enclosed tortuous arteries. This quantification was based on data from T2-weighted, pre-Ferumoxytol, and post-Ferumoxytol 2D GRE MR images. The utilization of both T2-weighted and 2D T2* GRE data aimed to address partial volume effects and suboptimal contrast inherent to T2* imaging. Among the entire subject group, a subgroup of 19 individuals underwent an improved imaging protocol that included a pre-Ferumoxytol 2D-GRE sequence to better visualize the PVS-tortuous artery complexes and better identify small arteries.

Two radiologists independently identified the tortuous small vessels and manually drew the vessel of interests (VOIs) without any knowledge of the participants' age and BMI to ensure unbiased assessments for the reliability evaluation.

### Statistical Analysis

Statistical analyses were conducted using SPSS software (IBM, version 28.0). Participants were initially categorized into five subgroups based on age decades (21-30, 31-40, 41-50, 51-60, and >60 years old). To assess differences in tortuous artery numbers and establish age cutoffs, one-way analysis of variance (ANOVA) analysis was conducted. Significance was set at p < 0.05, with Tukey's test for multiple comparison correction. For quantitative tortuosity measurements (tortuous artery numbers, TI, BL, and cw-ICM), young (age ≤ 50, n = 30) and old (age > 50, n = 7) groups were compared using independent t-tests. We then applied Bonferroni correction to account for multiple comparisons, with significance set at p<0.0125 adjusting for 4 tests. All datasets were tested for normal Gaussian distribution using Shapiro-Wilk test. In cases where the dataset did not exhibit a Gaussian distribution, we applied the Kruskal-Wallis test followed by Dunn's multiple comparison test to determine significance. Fisher's exact tests were employed for categorical variables (sex, hyperlipidemia, diabetes, smoking, and alcohol). The relationship between the number of tortuous arteries and age was evaluated using quadratic regression. Partial correlation analyses were performed to evaluate the association between quantitative tortuosity measurements (TI, BL, and cw-ICM) and age, with TI, BL, or cw-ICM as dependent variables separately and age as the independent variable. These analyses included sex, BMI, BP, and other vascular risk factors as covariates. Similar analyses were conducted to examine the relationship between vascular tortuosity and brain tissue volume (fractional GM volume and WM volume). Multiple linear regression followed with Kolmogorov-Smimov normality test was used to determine the effects of age, sex, BMI, and vascular risk factors on tortuosity measurements. Univariate linear regression was used to explore the relationship between age and the number of PVS with enclosed tortuous arteries. Furthermore, intraclass correlation coefficients (ICCs) were calculated to evaluate the inter-rater reliability.

**Table 1:** Demographic Characteristics, Tortuosity Measurements, and Brain Tissue Volume of the Study Participants.

Characteristic	All Participants (n = 37)	Younger Group (n = 30)	Older Group (n = 7)	P value
**Age**	38 ± 14 (21-70)	32 ± 8 (21-50)	62 ± 7 (51-70)	< 0.001^*^
**Sex (F/M)**	19/18	13/17	6/1	0.09
**Systolic BP (mmHg)**	120 ± 13.4	119 ± 12.5	128 ± 8.7	0.14
**Diastolic BP (mmHg)**	72 ± 11.9	71 ± 12.5	76 ± 14.9	0.50
**BMI**	25.3 ± 4.8	25.5 ± 2.7	27.6 ± 4.2	0.28
**Hypercholesterolemia, n (%)**	5 (13.51%)	1 (3.33%)	4 (57.14%)	< 0.001^*^
**Diabetes, n (%)**	3 (8.11%)	0 (0%)	3 (42.86%)	< 0.001^*^
**Smoking, n (%)**	2 (5.41%)	1 (3.33%)	1 (14.29%)	0.25
**Alcohol, n (%)**	5 (13.51%)	3 (10%)	2 (28.57%)	0.20
**Number of tortuous arteries**	40.6 ± 34.2	29.2 ± 21.7	94.3 ± 31.1	< 0.001^*^
**TI**	1.11 ± 0.03	1.10 ± 0.03	1.14 ± 0.02	0.02
**BL (mm)**	0.36 ± 0.15	0.31 ± 0.11	0.60 ± 0.08	< 0.001^*^
**cw-ICM**	8.75 ± 3.11	7.89 ± 2.75	12.31 ± 1.79	< 0.001^*^
**Fractional GM ratio (%)**	49.37 ± 2.97	50.16 ± 2.48	46.01 ± 2.63	< 0.001^*^
**Fractional WM ratio (%)**	40.86 ± 1.69	41.08 ± 1.72	40.30 ± 1.37	0.27

Data are means ± SDs with ranges in parentheses. Significance was determined by an independent t-test followed by Bonferroni correction, with significance level set at p<0.0125 adjusting for 4 tortuosity measurement comparisons. The asterisk (*) denoted a significant difference. F = female, M = male, BP = blood pressure, BMI = body mass index, TI = tortuosity index, BL = bending length, cw-ICM = curvature weighted inflection count metric, GM = gray matter, WM = white matter.

## RESULTS

### Demographics, Vascular Tortuosity, and Brain Volumes

Based on the significant tortuous changes observed in MAs in the age decade of 51-60, we established a cutoff age of 50 to distinguish between young and old groups. Among 37 subjects (age = 38±14 [SD], 19 females), we identified 30 young subjects with ages ≤50 years old (age = 32±8 [SD], 13 females) and 7 old subjects aged over 50 (age = 62±7 [SD], 6 females). Selecting an age cutoff of 50 was based on the rationale that it would provide a more sensitive measure of early age-related small vessel changes compared to using a conventional age cutoff of 65. There were no significant differences in systolic and diastolic BP, and BMI between the young and old groups (p=0.14, p=0.50, and p=0.28, respectively). A notable trend was observed, with older individuals showing a significantly higher incidence rate of hyperlipidemia and diabetes compared to young subjects (p<0.001). There were no significant differences in smoking and alcohol consumption between old and young groups (p=0.25, p=0.2, respectively).

Older subjects showed a significantly greater number of tortuous arteries (94.3±31.1) than young subjects (29.2±21.7, p<0.001), even though tortuous MAs were observed in their twenties. Additionally, TI, BL, and cw-ICM were higher in the old group than that in the young group (p=0.02, p<0.001, and p<0.001, respectively). The young group exhibited a higher GM ratio (p< 0.001) compared to the old group. However, no significant difference was observed in terms of the WM ratio (p=0.27). Detailed demographic characteristics, tortuosity measurements, and tissue volumes are summarized in Table 1.

### Detection of small tortuous arteries on USPIO-Enhanced 7T MRI

**Figure 2.**
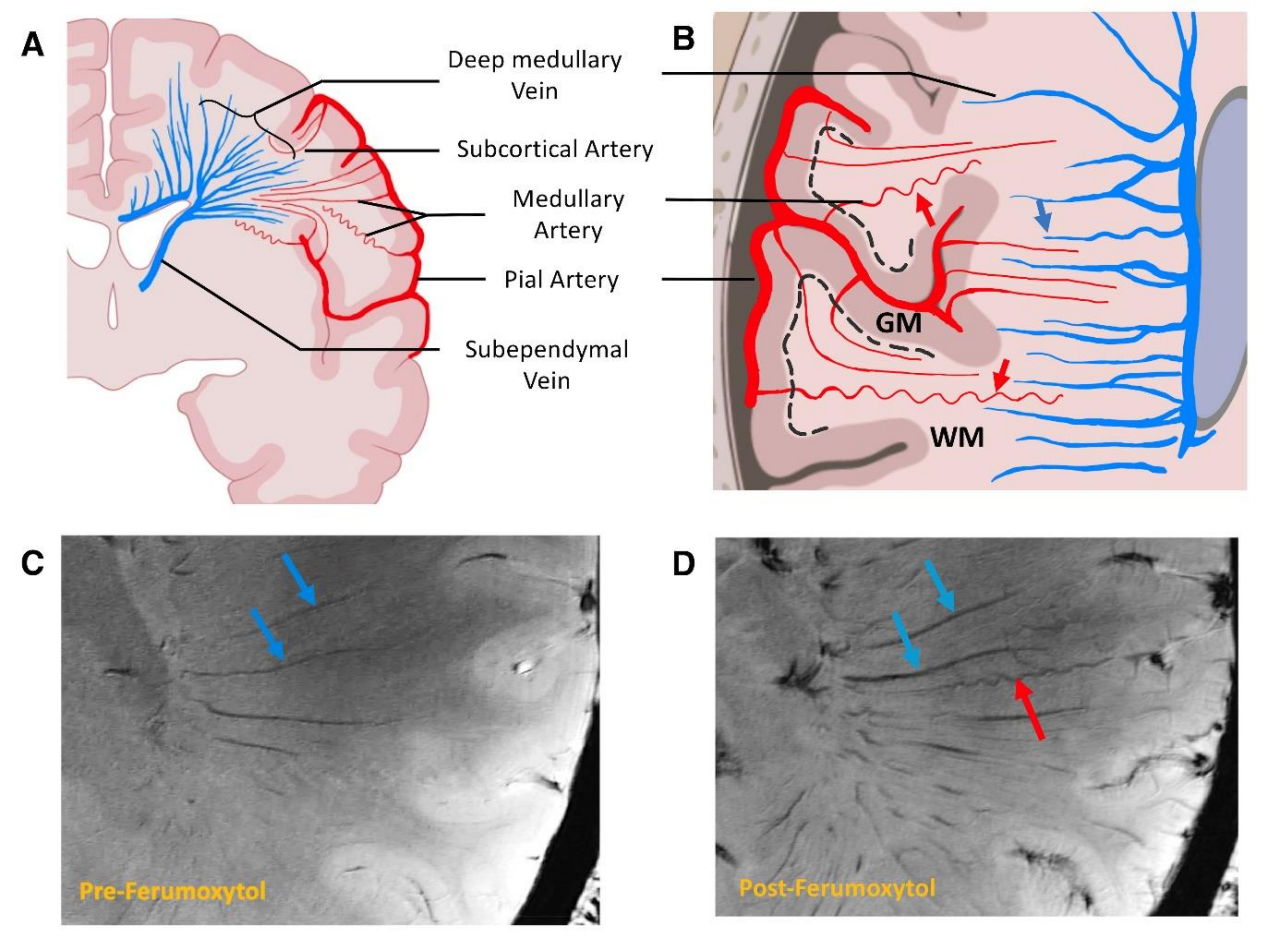
**Illustration of medullary arteries and veins**. (**A**) From the coronal view, medullary arteries (red) run linearly from the pial surface arteries to the lateral ventricle (LV). Medullary veins (blue) have a fan-shaped configuration and converge centrally to the lateral ventricle. (**B**) In the axial view, the arteries (red arrows) become abruptly tortuous as they penetrate the white matter (WM) and represent the corkscrew appearance. In contrast, medullary veins are less tortuous (blue arrow) with a parallel distribution along the LV and get thicker as they drain into the subependymal vein. The black dashed line indicated the border between gray matter (GM) and WM. In magnitude images of three-dimensional gradient dual-echo (3D-GRE) before (C) and after (D) the Ferumoxytol administration, the veins (blue arrows) were visible in both pre- and post-Ferumoxytol data; However, the artery (red arrow) was only visible after the Ferumoxytol administration and got thinner as they penetrate the WM. The vessel-tissue contrast was higher (darker) in veins than in arteries owing to a higher susceptibility baseline.

Consistent with histopathology studies [27], our results reveal that MAs originate from the superficial pial arteries, traverse through GM towards the subcortical WM, deep WM and lateral ventricle. Initially, MAs have a diameter of approximately 200μm, which arise from the pia artery and progressively reduce their size as they supply deep WM with few anastomoses. In contrast, medullary veins (MVs) drain blood from subcortical region and with increasing diameter towards lateral ventricle and converge with subependymal veins into deep venous system (Fig. 2A-B) [28]. Arterial Tortuosity becomes prominent after MAs traverse into the WM from cortical GM, likely due to the looser texture of WM [1]. MAs can be differentiated from MVs by the notable alteration in susceptibility contrast of arterial blood, which is visible exclusively in post-Ferumoxytol images. In contrast, MVs are visible on both pre-and post-contrast images (Fig. 2C-D). Compared to the MVs, MAs appear smaller in diameter with slightly less vessel-tissue susceptibility contrast on post-Ferumoxytol SWI. Additionally, we observed the tortuosity of MAs has a characteristic corkscrew appearance, whereas tortuous veins tend to have less curved courses, possibly due to inherent differences in their wall structures [29].

### Age and BMI Effects on the MA Tortuosity Measurements

**Figure 3.**
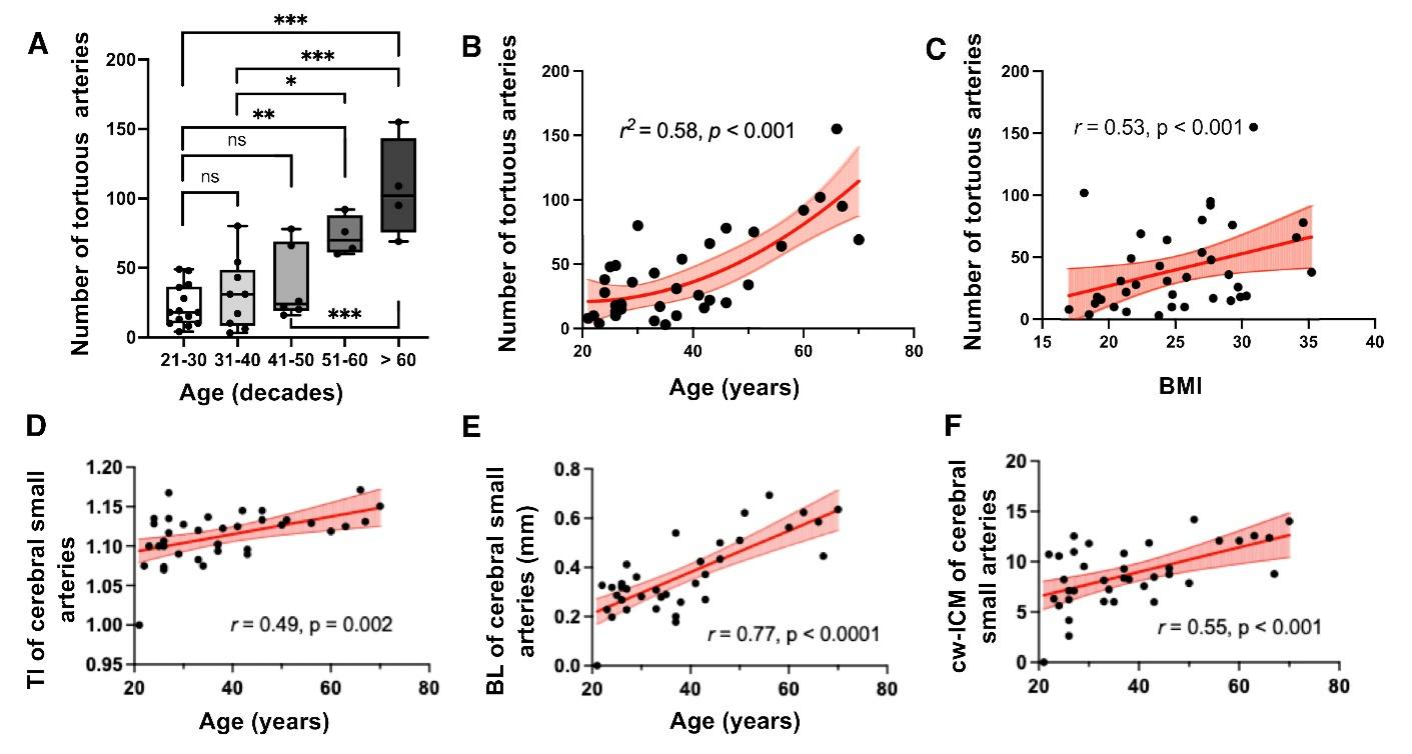
**Arterial tortuosity in association with age and BMI**. (**A**) One-way analysis of variance (ANOVA) revealed differences among different age decades regarding the number of tortuous arteries (21-30: 22.4±14.9 (n=14); 31-40: 30.6±25.3 (n=9); 41-50: 38±26.8 (n=6); 51-60: 73±14.4 (n=4); >60: 107±36.0 (n=4)). Significant differences initiated from the age of fifties compared to 21-30 and 31-40 age groups (p=0.003, and p=0.03, respectively). P values were corrected for multiple comparisons using Tukey’s test. (**B**) There was a quadratic non-linear increase in the tortuous arteries number with age (r^2^=0.58, p<0.001. Number of tortuous arteries = 0.037*Age2-1.458*Age+35.34). (**C**) After adjusting for age, gender, and systolic/diastolic blood pressure (BP), body mass index (BMI) was associated with the number of tortuous arteries (r=0.53, p<0.001). After adjusting for BMI, gender, and blood pressure, there were significant positive correlations between (D) tortuosity index (TI), (E) bending length (BL), and (F) curvature-weighted inflection count matric (cw-ICM) of the cerebral medullary arteries and age (r=0.47, p=0.003; r=0.64, p<0.001; r=0.54, p<0.001, respectively). Significance was set at a p-value less than 0.05, *p<0.05, **p<0.01, ***p<0.001.

The number of small tortuous arteries on Ferumoxytol-enhanced T2*-weighted images increases with age. After multiple comparison correction, one-way ANOVA analyses revealed a significantly greater number of tortuous arteries in the fifties age range versus the age decade of twenties (p=0.003) and thirties (p=0.03) (Fig. 3A). These findings suggest that significant vascular remodeling may begin around the age of 50 years. After adjusting for sex, BP (systolic and diastolic), and BMI as covariates, the number of tortuous arteries exhibited a quadratic non-linear relationship with age (*r^2^*=0.58, p<0.001, tortuous artery number=0.037*age^2^-1.458*age +35.34) (Fig. 3B).

Partial correlation analyses revealed positive relationships between age and TI (*r*=0.49, p= 0.002), BL (*r*=0.77, p<0.0001), and cw-ICM (*r*=0.55, p<0.001) after controlling sex, BMI, BP, and other vascular risk factors as covariates, which indicated increased tortuosity of cerebral small arteries with aging (Fig. 3D-F). Furthermore, the number of tortuous MAs exhibited a significant positive correlation with BMI after adjusting for age, sex, BP, and other vascular risk factors (*r*=0.53, p<0.001) (Fig. 3C). Partial correlation results suggested BMI positively related with TI (*r*=0.37, p=0.02), and cw-ICM (*r* = 0.36, p=0.02) after adjusting covariates. There was no significant correlation between BMI and BL (*r* = 0.25, p=0.13). Multiple linear regression analyses indicated that vascular tortuosity could be determined by age, BMI, and vascular risk factors including hypercholesterolemia and smoking, as summarized in Table 2.

**Figure 4.**
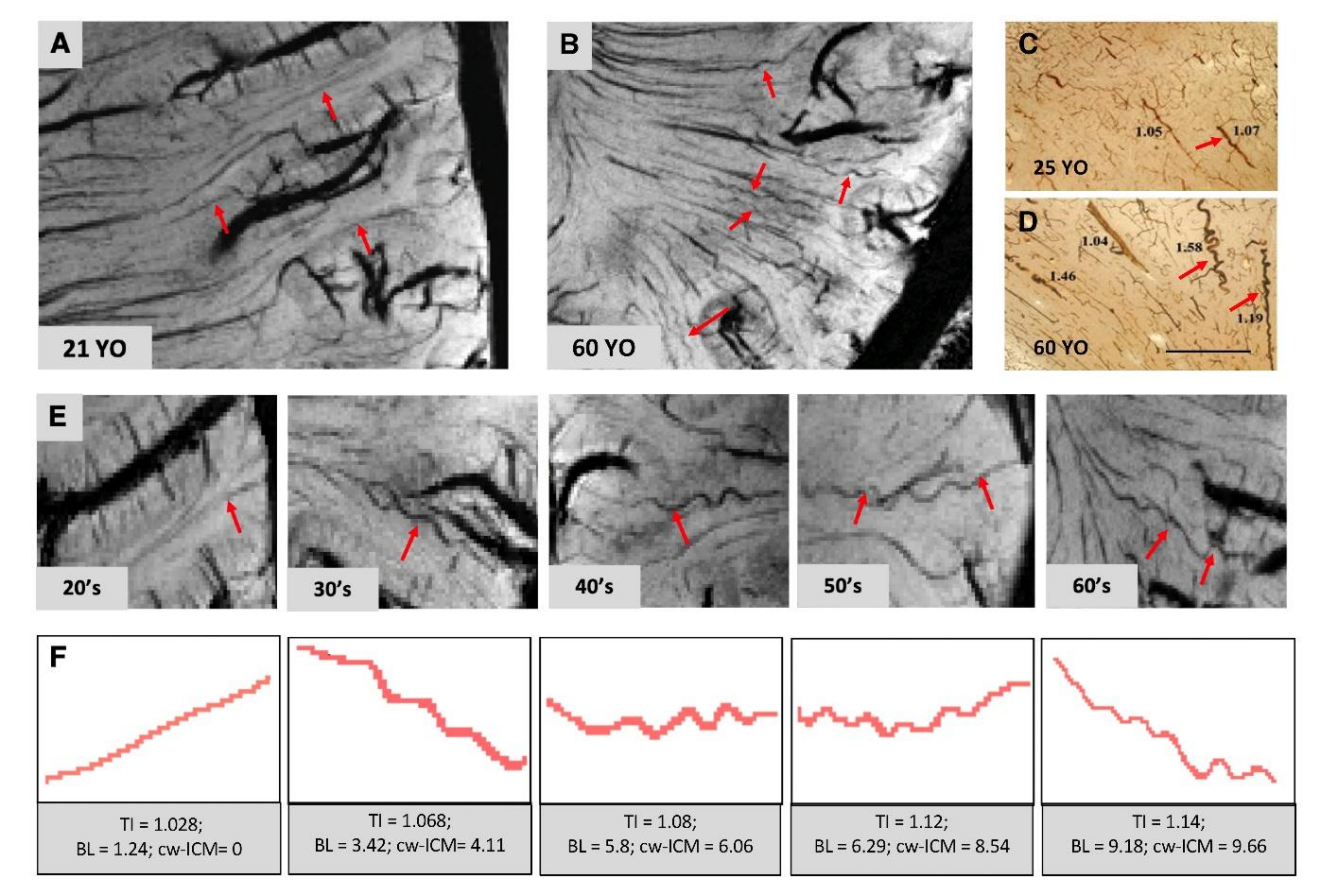
**Three-dimensional gradient-echo (3D-GRE) MRI after contrast greatly improved the detection of cerebral small arteries**. (**A**) In a 21-year-old (YO) young subject, most of the small arteries revealed by the post-contrast 3D susceptibility-weighted imaging (SWI) with minimum intensity projection of four (mIP=4) were straight; (B) The old subject (60 YO) had more tortuous cerebral small arteries. Alkaline phosphatase-stained sections showed that compared with the young subject (25 YO) (C), small arterioles in the deep white matter (WM) were more tortuous in the old subject (60 YO) (D) (C and D were reprints from Thore et al., (2007)). (**E-F**) Artery segments (red arrows) and measurements of different age decades from 3D-GRE data showed increasing tortuosity level measured with tortuosity indices in cerebral small arteries, which aligned with histological findings.

Additionally, illustrative post-Ferumoxytol SWI images directly showed increased tortuosity of MAs in older subjects compared to young subjects (Fig. 4A-B), consistent with findings (Fig. 4C-D) reported by previous pathohistological study [4]. Representative subjects from each age decade demonstrated a progressive increase in tortuosity severity (Fig. 4E) or tortuosity measurements with age (Fig. 4F). The tortuosity measurements obtained from two raters showed the following intraclass correlation coefficient (ICCs) - TI: ICC = 0.87; 95%CI = 0.76, 0.93; p<0.001; BL: ICC = 0.94; 95%CI = 0.86, 0.97; p<0.001; cw-ICM: ICC = 0.92; 95%CI = 0.86, 0.96; p<0.001).

### Relationships Between MA Tortuosity and Brain Tissue Volume

Partial correlation result showed a significant negative association between the number of tortuous arteries and WM ratio (*r*=-0.488, p=0.011) after adjusting age, sex, BMI, and BP; whereas we did not observe associations between TI, BL, and cw-ICM and WM ratio (*r*=0.27, p=0.2; *r* =-0.07, p=0.75; *r*=0.07, p=0.73, respectively). In terms of GM ratio, no significant results were observed in relation to the number of tortuous arteries, TI, BL, or cw-ICM (*r*=0.019. p=0.93; *r* =0.017, p=0.94; *r*=-0.031, p=0.88; *r*=-0.1, p=0.644).

**Table 2:** Multiple Linear Regression of Different Tortuosity Measurements.

Characteristic	Tortuous Artery Numbers		TI		BL		cw-ICM	
	coeff	P value	coeff	P value	coeff	P value	coeff	P value
**Age (year)**	0.53	< 0.001*	0.44	<0.001*	0.69	<0.001*	0.28	0.04*
**Sex**	-0.45	< 0.001*	0.03	0.86	0.04	0.68	-0.07	0.56
**BMI**	0.34	0.003*	0.42	0.02*	0.32	0.003*	0.42	0.002*
**Systolic BP (mmHg)**	0.14	0.42	-0.13	0.65	0.13	0.42	0.25	0.22
**Diastolic BP (mmHg)**	-0.03	0.85	0.20	0.37	-0.19	0.13	0.05	0.75
**Hypercholesterolemia**	-0.14	0.26	0.24	0.21	0.26	0.02*	0.47	0.002*
**Diabetes**	0.18	0.18	-0.30	0.18	-0.16	0.20	-0.09	0.56
**Smoking**	0.05	0.66	0.11	0.52	0.21	0.04*	0.40	0.002*
**Alcohol**	-0.02	0.83	-0.06	0.73	0.24	0.02*	0.06	0.66
**Adjusted R2**	0.71		0.26		0.76		0.62	
**P value**	< 0.001*		0.04*		<0.001*		<0.001*	

Adjusted *R*^2^ was determined by multiple linear regression with significance level set as p<0.05. coeff = coefficient, TI = tortuosity index, BL = bending length, cw-ICM = curvature weighted inflection count metric. The asterisk (*) denoted a significant difference.

### Relationships Between MA Tortuosity, White Matter Hyperintensities, and Perivascular Space

**Figure 5.**
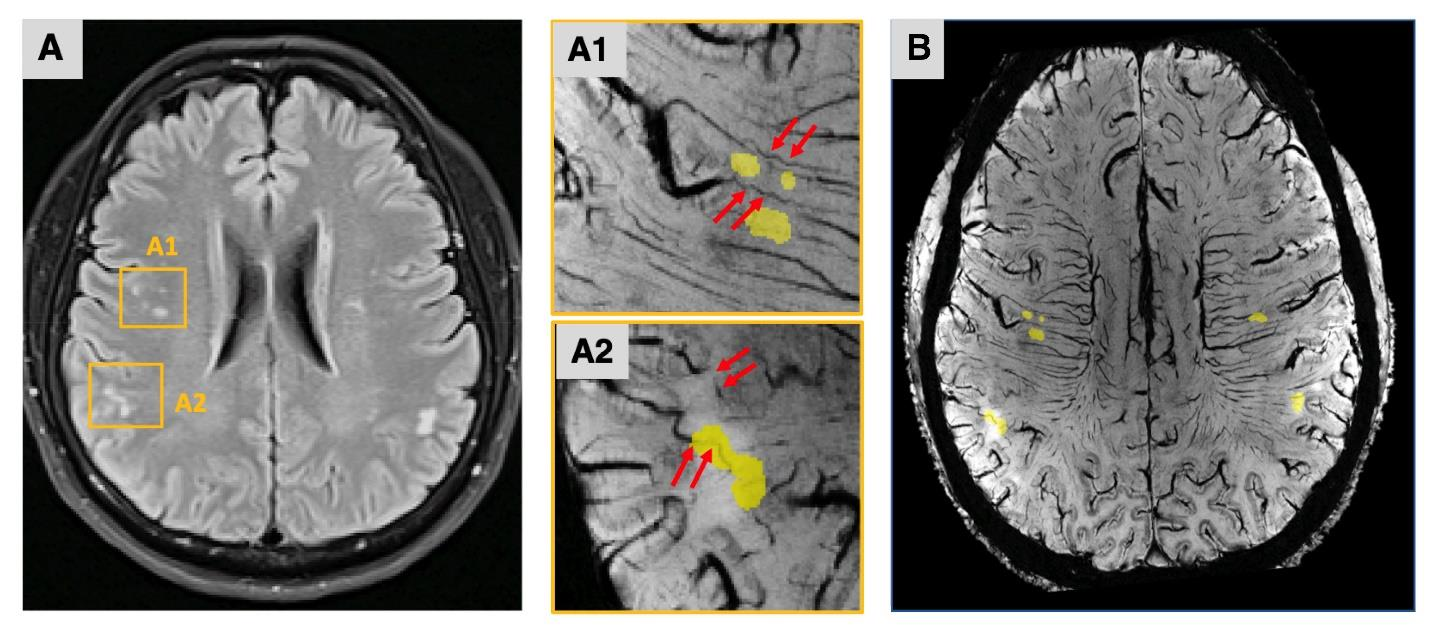
**Spatial relationship between tortuous arteries and white matter lesions**. (**A**) White matter lesions were revealed on FLAIR. A1 and A2 were magnified panels of lesions overlaid on the post-contrast 3D susceptibility-weighted imaging (SWI) data. The WM lesions (yellow masks) were adjacent to or at the distal end of the tortuous arteries (red arrows). (**B**) After linear registration, the lesion mask was overlaid on the post-contrast SWI (mIP = 2) images. The ischemic leukoaraiosis located in areas where the cerebral small arteries (i.e., medullary arteries) provide the blood supply.

Subcortical WM lesions were revealed on FLAIR MRI (Fig. 5A). We overlaid the FLAIR lesion map onto the post-contrast 3D-SWI (mIP = 2) data using linear registration (Fig. 5B). Our observation indicated a spatial relationship wherein the WM lesions were proximity to the tortuous arteries or situated at the distal end of these tortuous arteries (Fig. 5A1-A2). These arteries did not display a branching pattern or anastomotic connections. Furthermore, after reviewing the FLAIR images containing WMHs, we discovered that this spatial relationship is notably pronounced in deep WM or subcortical WM instead of periventricular region. This discrepancy could potentially signify their distinct underlying pathogenesis.

**Figure 6.**
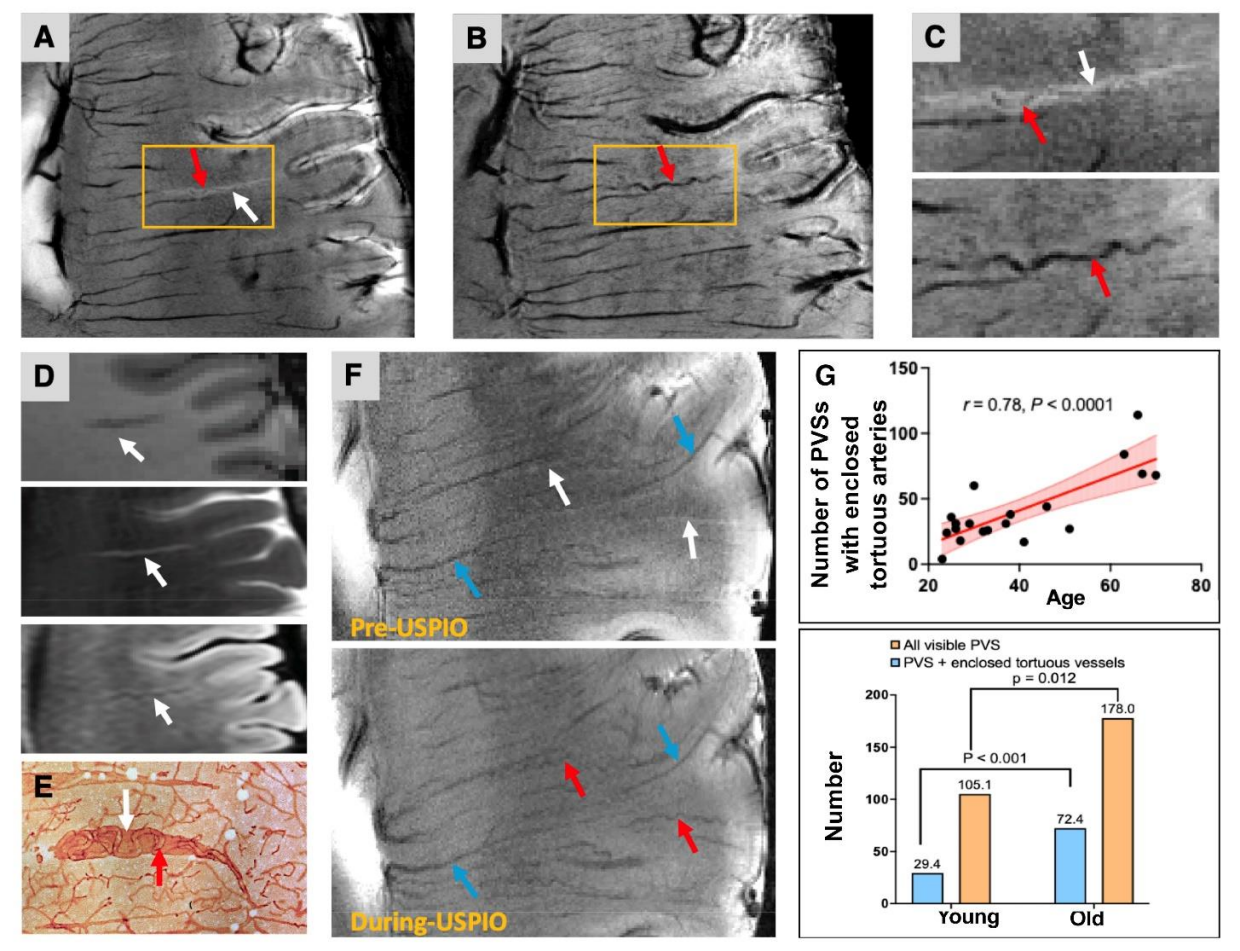
**With Ferumoxytol, the 2D gradient echo (2D-GRE) data (A) and 3D susceptibility-weighted imaging (SWI) data (B) revealed a perivascular space (PVS) and the enclosed tortuous artery**. Unless otherwise noted, arteries, veins, and PVS are indicated by red, blue, and white arrows. (**C**) Magnified view of yellow boxes in the 2D GRE (up) and post-contrast SWI (bottom). (**D**) Perivascular space (PVS) in the conventional MRI (i.e., T1-weighted (left), T2-weighted (middle), and fluid-attenuated inversion recovery (FLAIR) MRI (right)). (**E**) A thick celloidin section stained with the antibody to collagen IV showed tortuous arterioles screwed in the cavities. (E was reprinted from Brown et al., (2002)). (**F**) Linear hyperintense PVSs (white arrows) and veins (blue arrows) were revealed in the pre-USPIO 2D-GRE MRI. With USPIO, enclosed tortuous arteries (red arrows) could be detected. The PVSs were less evident in the image data with USPIO, which might be attributable to the susceptibility blooming effects induced by the contrast agent. (**G**) The number of PVSs with enclosed tortuous arteries was positively associated with age (n = 19, r=0.78, p<0.0001). Compared with young subjects (n=14), the old subjects (n=5) had more PVS (p=0.012), more PVS with tortuous arteries enclosed (P <0.001). The ratio between PVS with enclosed tortuous arteries and total visible PVS was higher in old subjects (40.7%) versus young subjects (28%). Significance was determined using an independent t-test followed by Bonferroni correction. Significance level was set at p<0.025 adjusting for 2 tests.

At 7T, axial 2D GRE sequence provided unique T2 and T2* contrasts for detecting vessels, surrounding tissues, and PVS with CSF. Example illustrations in Figure 6 show tortuous small arteries (hypo-intensity) surrounded by dilated PVS (hyperintensity) observed from the 2D GRE T2* data (Fig. 6A, 6C). While the 3D GRE data better delineated the small artery (Fig. 6B), PVS visualization was limited. Conventional MRI revealed thin linear or oval-shaped PVS (Fig. 6D) without detailed morphology. High-spatial-resolution MR imaging findings aligned with histological staining, showing elongated tortuous arteriole twisted within the enlarged PVS cavities (Fig. 6E) [29]. In some cases, the pre-contrast 2D-GRE data underestimated the PVS, whereas the enclosed tortuous arteries could be visualized on post-contrast images (Fig. 6F). Univariate linear regression demonstrated a positive association between the number of PVSs with enclosed tortuous arteries and age (*r*=0.78, p<0.0001). Compared to the young group, the old group had more PVS (p=0.012) and more PVS with enclosed tortuous arteries (p<0.001) as well as a higher ratio of PVS with enclosed tortuous arteries to total PVS numbers (young: 28% versus old: 40.7%) (Fig. 6G).

## DISCUSSION

Small vessel disease (SVD) is pivotal in various neurovascular and neurodegenerative disorders [1, 30]. While conventional SWI effectively detects small veins with higher deoxygenated blood, the visualization of small artery abnormalities using conventional imaging techniques, including gadolinium-enhanced MRI, remains challenging due to limited vessel contrast and resolution. In this study, we investigated small cerebral arteries (50-200μm) and age-related tortuosity using Ferumoxytol-enhanced 7T MRI by taking advantage of the blooming effect and susceptibility shift. Notably, this in vivo study unveiled corkscrew-like small arteries, previously observed solely in histopathological studies. These changes emerge at a younger age than conventionally anticipated and escalate with increasing age and BMI. This dependency provides valuable insights into the role of vascular degeneration in SVD, tissue loss, and WMHs [3, 31].

Age-related tortuosity in large arteries have been well studied [32, 33]. However, the in vivo detection of small vessel abnormalities, especially in individuals at the preclinical stage, remains largely unexplored. WMHs have been conventionally used as an indirect sign for age-related CSVD without revealing the actual small vessel changes. With aging, microvascular abnormalities that are undetectable with clinical imaging, such us tortuosity, proceed silently long before clinical manifestation. Over decades, the flow pulsation renders small arteries, characterized by higher resistance and thinner walls, susceptible to mechanical stress, leading to morphological changes (e.g., corkscrew). These tortuous MAs disrupt the blood flow and oxygen delivery to the subcortical and deep WM, culminating in WM atrophy, ischemia and WMHs formation [34, 35].

The number of tortuous MAs was observed to correlate with WM atrophy rather than GM atrophy. This linkage can be attributed to the compromised cerebral blood flow (CBF) resulting from tortuous MAs, underscoring the importance of detecting arteries in vivo. Simultaneously, the induced cerebral hypoperfusion can contribute to ischemic damage and formation of WMHs. Indeed, our utilization of Ferumoxytol-enhanced 7T MRI revealed compelling evidence through which we exposed the spatial relationship between the tortuous small arteries and WMHs at a microscopic level, as illustrated in Figure 5. This relationship was prominent in deep WMHs (DWMLs) rather than periventricular WMHs (PVWMLs), which highlighted their different histopathology and etiology. Few in vivo imaging investigations possess the capability to directly discern these alterations. PVWMLs could be linked to the Wallerian degeneration and have been considered predictive of border zone infarcts, especially around the posterior ventricle horns [36, 37]. On the other hand, DWMLs are more likely to ischemic and associated with lacunar infarcts and SVD [38]. As shown in Figure 4, our observations revealed that the distinctive corkscrew appearance of small arteries occurred in adults in their 30s, and there was a notable and statistically significant increase in the number of these tortuous arteries after the age of 50. This finding suggests that vascular contributions to SVD might initiate much earlier in life than previously anticipated. These changes in vascular tortuosity are evidently silent in younger individuals, only becoming significant at some point in later life. As demonstrated by Thore et al. [4] in their comprehensive postmortem study, encompassing a wide age range and not limited to dementia cases, blood vessels displaying a prominently high curl score were not observed before the fifth decade. Hassler et al. [39] investigated the brains from individuals with dementia, employing a micro-angiographic method. They observed the microvascular loop formation in brains affected by senile dementia in individuals over forty years old, even before reaching out our cutoff age in this study.

Equally noteworthy, we observed higher tortuosity of small arteries in participants with elevated BMIs, a trend seemingly unrelated to age. This implies that these morphological changes in small arteries are linked to cardiovascular risk factors, potentially mitigated through lifestyle adjustments or early medical intervention. Previous studies have indicated that chronic low-grade inflammation and lipid accumulation induced by excessive body weight can accelerate vascular wall remodeling, and further exacerbate perfusion dysfunction [40]. Further in vivo investigations are needed to validate the clinical significance of these findings.

Histopathological insights into tortuous middle cerebral arteries (MAs) within the elderly population have been garnered through postmortem investigations. These insights have revealed correlations with collagen deposition, wall thickening, and lumen constriction [1, 41]. However, these studies suffered from the limitations of end-stage disease and tissue fixation damage, thus lacking the clinical utility necessary for disease monitoring and prevention strategies. Our results align closely with the histopathological findings presented in studies where alkaline phosphatase staining was employed [4, 29]. These results demonstrated that the tortuosity index typically resided within the range of approximately 1.1 to 2.5, thus underscoring its noteworthy significance in neurodegenerative disorders. MAs characterized by histologically reported diameters ranging from 50 to 200 µm were effectively discernible through imaging techniques encompassing broad brain coverage, a capability surpassing the confines of alkaline phosphatase staining. Postmortem assessments, by their nature, tended to underestimate vessel size due to the tissue's 15-20% reduction in size upon fixation and lack of blood flow [42]. Earlier investigations [15] demonstrated that Ferumoxytol-enhanced SWI exhibited the capability to identify diminutive arteries with diameters ranging from 50 to 100 µm using a Ferumoxytol dose of 2 mg/kg, echo time (TE) of 15ms, and a voxel aspect ratio of 1:4 (in-plane to through-plane, i.e., 0.25×0.25×1 mm^3^) [15, 17], the same as used in the current study. These findings unequivocally validate the feasibility and reliability of small vessel detection.

In this study, we employed multiple methods to distinguish small arteries from veins. First, arteries rich in oxygenated hemoglobin remain invisible in pre-Ferumoxytol SWI (unlike veins); however, they become discernible in post-Ferumoxytol SWI. Second, from an anatomical standpoint, MAs act as terminal arteries originating from the pial surface of the brain, traversing the cortical layer to supply the deeper WM. In contrast, MVs at the periventricular level run parallel to MAs, increasing in diameter as they converge and drain into the ependymal veins. Third, the presence of corkscrew geometries of MAs, characterized by multiple inflection points, is a trait predominantly observed in small arteries rather than veins. This can be attributed to the different pulse pressures, flow patterns, and wall structures between these two vessel types [4, 43]. Lastly, with a higher susceptibility baseline, veins exhibited a slightly thicker diameter and darker intensity on Ferumoxytol-enhanced SWI owing to the superimposed susceptibility effects.

Recent studies have underscored the significance of PVS in relation to blood-brain barrier dysfunction, waste clearance, and cognitive decline [44-46]. Moreover, these studies have observed an escalation in PVS quantities among the elderly population. In our present study, we not only identified an augmentation in the number of PVS but also noted an increase in PVS-tortuous artery complexes with advancing age. This observation gives rise to a pivotal inquiry regarding the chronological sequence of these processes. Our proposition is that the genesis of tortuous vessels and dilated PVS constitutes a dynamic progression. It is initiated by the elongation of tortuous vessels, which exert outward pressure, leading to the expansion of PVS cavities. Subsequently, the enlarged PVS creates an environment conducive to the further development of tortuosity. However, it's worth noting that not all tortuous arteries were accompanied by an encircling PVS. This discrepancy could potentially be attributed to the partial volume effect of cerebrospinal fluid (CSF), the diminutive size of the arteries, and even vessel occlusion as elucidated in the histopathological investigation [29]. For future studies, refining spatial resolution and augmenting the USPIO dose in both T2- and T2*-weighted imaging modalities might facilitate a more comprehensive characterization of their interrelationship.

Our study had several limitations. First, the axial imaging plane used in this study is well-suited for capturing MAs, yet it is unsuitable for lenticulostriate arteries originating from the Willis circle arteries level (i.e., oriented in the foot-to-head direction). Second, the dosage of Ferumoxytol administered in our study (2mg/kg) was comparatively smaller than the 4 mg/kg dosage employed in other Ferumoxytol studies[15, 17], which may potentially curtail our capacity to differentiate minute tortuous vessels within PVS. Third, our image processing involved several manual steps which potentially introduce variability and reduced reproducibility. To mitigate the variability in quantitative tortuosity assessment, we implemented the following procedures: 1) Maintained a consistent FOV position to keep uniform brain coverage; 2) Defined tortuous arteries as those exhibiting five or more turns to ensure different raters use the same criteria when counting the tortuous artery numbers; 3) Selected a slab of 3D-SWI image to guarantee a thorough coverage and reliable quantitative measurements; 4) Conducted automated centerline tracking and quantitative analyses to minimize the need for further manual intervention. Lastly, there was a lack of clinical data (e.g., glucose, lipid levels) on some cardiovascular risk factors associated with tortuosity, and the sample size was relatively small. Notwithstanding these limitations, the primary intent of this study was to establish proof-of-concept. Through the utilization of USPIO-enhanced MRI, we aimed to demonstrate the capability of detecting small arterial tortuosity at a micro-level, thereby enhancing comprehension of its involvement in age-related cognitive decline.

In summary, our study demonstrates the potential of Ferumoxytol-MRI at 7T to visualize age-related small artery tortuosity at a micro-level, a critical stage influencing vascular health and the onset of CSVD. This non-invasive method offers timely monitoring and identification of these age-related alterations *in vivo*, enhancing our understanding of SVD's origins. Furthermore, this advancement holds promise in targeted therapeutic strategies for preventing cognitive decline in the elderly.
